# Effect of Del Nido cardioplegia on ventricular arrhythmias after cardiovascular surgery

**DOI:** 10.1186/s12872-020-01844-z

**Published:** 2021-01-13

**Authors:** Chang Shu, Liang Hong, Xiao Shen, Wenhao Zhang, Yongsheng Niu, Xiaochun Song, Jie Kong, Cui Zhang

**Affiliations:** 1 Department of Intensive Care Unit, Nanjing First Hospital, Nanjing Medical University, 68 Changle Road, Nanjing, 210006 Jiangsu Province China; 2 Department of Interventional Radiology, Nanjing First Hospital, Nanjing Medical University, 68 Changle Road, Nanjing, 210006 Jiangsu Province China

**Keywords:** Del nido, Cardioplegia, Cardiopulmonary bypass, Arrhythmia, Myocardial damage

## Abstract

**Background:**

Del Nido cardioplegia (DNC) has been proven safe and effective in pediatric patients. However, the use of DNC in adult undergoing cardiovascular surgery lacks support with substantial evidence. This study aimed to evaluate the efficacy of DNC as a cardioplegia of prophylaxis to ventricular arrhythmias associated to cardiovascular surgery in adult patients.

**Methods:**

This study recruited nine hundred fifty-four patients who underwent cardiopulmonary bypass surgeries in Nanjing Hospital affiliated to Nanjing Medical University between January 2019 and December 2019. Among 954 patients, 324 patients were treated with DNC (DNC group), and 630 patients were treated with St. Thomas cardioplegia (STH group). The incidence of postoperative arrhythmia as well as other cardiovascular events relavant to the surgery were investigated in both groups.

**Results:**

In DNC group, the incidence of postoperative ventricular arrhythmias was lower (12.4% vs. 17.4%, *P* = 0.040), and the length of ICU stay was shorter (1.97 ± 1.49 vs. 2.26 ± 1.46, *P* = 0.004). Multivariate logistic regression demonstrated that the use of DNC helped to reduce the incidence of postoperative ventricular arrhythmias (adjusted odds ratio 0.475, 95% CI 0.266–0.825, *P* = 0.010). The propensity score-based analysis and subgroup analysis indicated that DNC has the same protecting effects towards myocardial in all kinds of cardiopulmonary bypass surgeries.

**Conclusions:**

Del Nido cardioplegia may potentially reduce the incidence of postoperative ventricular arrhythmias, shorten the length of ICU stay and improve the overall outcome of the patients undergoing cardiovascular surgery.

## Background

Cardiac cardioplegia plays an essential role in cardiopulmonary bypass (CPB) to keep the beating heart at rest and create a reversible bloodless surgical field of vision for surgeons [1]. Different countries chose different types of cardioplegia. St. Thomas’s solution is most commonly used in Europe, Australia, New Zealand, and South America (63.6%,67.1% and 56.6% respectively) [2]. DNC is a cold blood cardioplegia modified with a higher level of potassium. It was initially developed by Professor Pedro Del Nido at the University of Pittsburgh given the use in children's immature myocardium [3]. Beyond the use in pediatrics, multiple animal experiments and clinical trials have proven that DNC can also excellently protect the mature myocardium [4–7].

Despite the findings mentioned above, the use of DNC in adult patients is currently limited. Stronger clinical evidence is required in order to generalize the use of DNC in the field of postoperative myocardio protection in adult patients. The Cleveland Clinic recommended the use of Del Nido solution in valve surgeries only but not in coronary artery bypass surgeries in considering its vague effects in protecting myocardium from ischemia [8].

Therefore, we conducted this retrospective study including adult patients who underwent all cardiovascular surgeries via CPB in Nanjing First Hospital affiliated to Nanjing Medical University during the year of 2019, and evaluated the effect of DNC solution on the incidence of arrhythmia and myocardial protection in protecting myocardium from postoperative arrhythmia.

## Methods

### Objects

This was a retrospective observational study, including post cardiovascular surgical patients who were admitted to the Cardiovascular Intensive Care Unit (ICU) department of Nanjing First Hospital (Nanjing Medical University) between January and December 2019.

### Enrollment

Patients who met the following criteria during the study period were highlighted and recruited as study objects: ① age > 18 years old and < 80 years old; ② received conventional cardiovascular surgery, including but not limited to heart valve replacement, valve repair, coronary artery bypass, etc.; ③ used cardiopulmonary bypass support; ④ used either DNC or STH solution as cardioplegia; ⑤ competent to provide informed consent or having a legally authorized principal to provide consent when he/she was not competent. Exclusion criteria: ① Off-pump coronary artery bypass graft (OPCAB); ② Intraoperative death; ③ Data insufficiency. Criteria of data shedding: For patients who met the criteria, due to incomplete medical records, laboratory examination data such as biochemical and myocardial enzymes, or changes in experimental reagents, they cannot be compared with other patients simultaneously. The study protocol was conducted by the Declaration of Helsinki and was approved by Ethics Committee of Nanjing First Hospital, Nanjing Medical University (KY20170811-03).

According to the type of cardioplegia used in cardiac surgery, patients were distributed into conventional St. Thomas’s cardioplegia group (STH group) and Del Nido cardioplegia group (DNC group).

### Clinical and laboratory measurements

1. Perioperative variables: Baseline characteristics including age, past medical history, Acute Physiology and Chronic Health Evaluation II (APACHE II), European system for cardiac operative risk evaluation score(euroSCORE), left ventricular ejection fraction (EF), biochemical indicators, data of ECG on admission and other perioperative variables were collected and recorded.
2. Surgical variables and grouping: We also collected the patients’ intraoperative data according to the medical record data such as surgery type, duration of operation, time of CBP, time of aortic occlusion (AB), and type of cardioplegia. After that, the patients were grouped according to type of cardioplegia.
3. Postoperative variables: Postoperative variables of the two groups of patients were also collected, including postoperative biochemical indicators: maximum serum potassium and creatinine within 24 h; postoperative cardiac enzymes within 24 h: troponin T (TnT), troponin I (TnI), creatine kinase (CK), creatine kinase isoenzyme (CK-MB), N-terminal forebrain natriuretic peptide precursor (NT-proBNP); left ventricular ejection fraction (EF) within 48 h, incidence of atrial fibrillation, incidence of ventricular arrhythmia and the length of ICU stay.

### Endpoint

1. Primary endpoints: adverse cardiac events which affect the hemodynamic stability and require medical intervention. Adverse cardiac events include atrial fibrillation, multifocal premature beat, R on T, ventricular tachycardia, ventricular fibrillation and other ventricular arrhythmias that was concerned and then treated by clinicians.
2. Secondary endpoints: length of ICU stay.

### Treatment

Case-specific treatments including amiodarone, metoprolol, lidocaine, as well as other antiarrhythmics or electroversion were given by on-call clinicians following the clinical judgements of the physician.

### Statistical analysis

Measurement data conforming to a normal distribution were described as mean (standard deviation). Independent sample t-tests (two-sided) were used for inter-group comparisons. Measurement data not conforming to normal distribution were expressed as median (lower quartile-upper quartile). Wilcoxon rank-sum tests were used for inter-group comparisons. The enumeration data were expressed in percentage and compared by the Pearson $\chi$^2^ test (two-sided). Fisher’s exact test was used when the expected frequencies of one or more cells were less than 5. Multivariate logistic regression analysis was performed with adjusted odds ratios (aORs) of 95% confidence intervals (95% CIs) to calculated the estimated association between observed clinic index and post-cardiac surgery arrhythmias. Prior to this process, continuous data were categorized on the basis of median values or widely recognized cut-off value. Propensity score matching (PSM) was used to reduce the surgical procedures’ bias(including operation type, operation times, AB time and CBP time). The propensity score was computed by logistic regression model based on surgical procedures, then subjects were 1:1 matched by the logit of estimated propensity score using the nearest neighbor matching algorithm with a caliper of 0.05, using “MatchIt” package in R 3.6.2 environment. The other statistical analyses were performed with SPSS version 18.0 (SPSS Inc., Chicago, USA). The values were considered to be statistically significant at *P* < 0.05.

## Results

A total of 1132 patients who underwent cardiac surgery admitted to Cardiovascular ICU of Nanjing First Hospital, Nanjing Medical University, from January 2019 to December 2019 were screened for potentially enrollment. The excluded cases were as follows: 82 cases of data loss, 64 patients did not use cardiopulmonary bypass support, 9 cases were younger than 18 years old, and 23 patients did not use Del Nido or St. Thomas’s cardioplegia. Eventually, a total of 954 patients were enrolled in the study and were divided into Del Nido cardioplegia group (DNC group, n = 324) and St. Thomas’s cardioplegia group (STH group, n = 630).

**Fig. 1:**
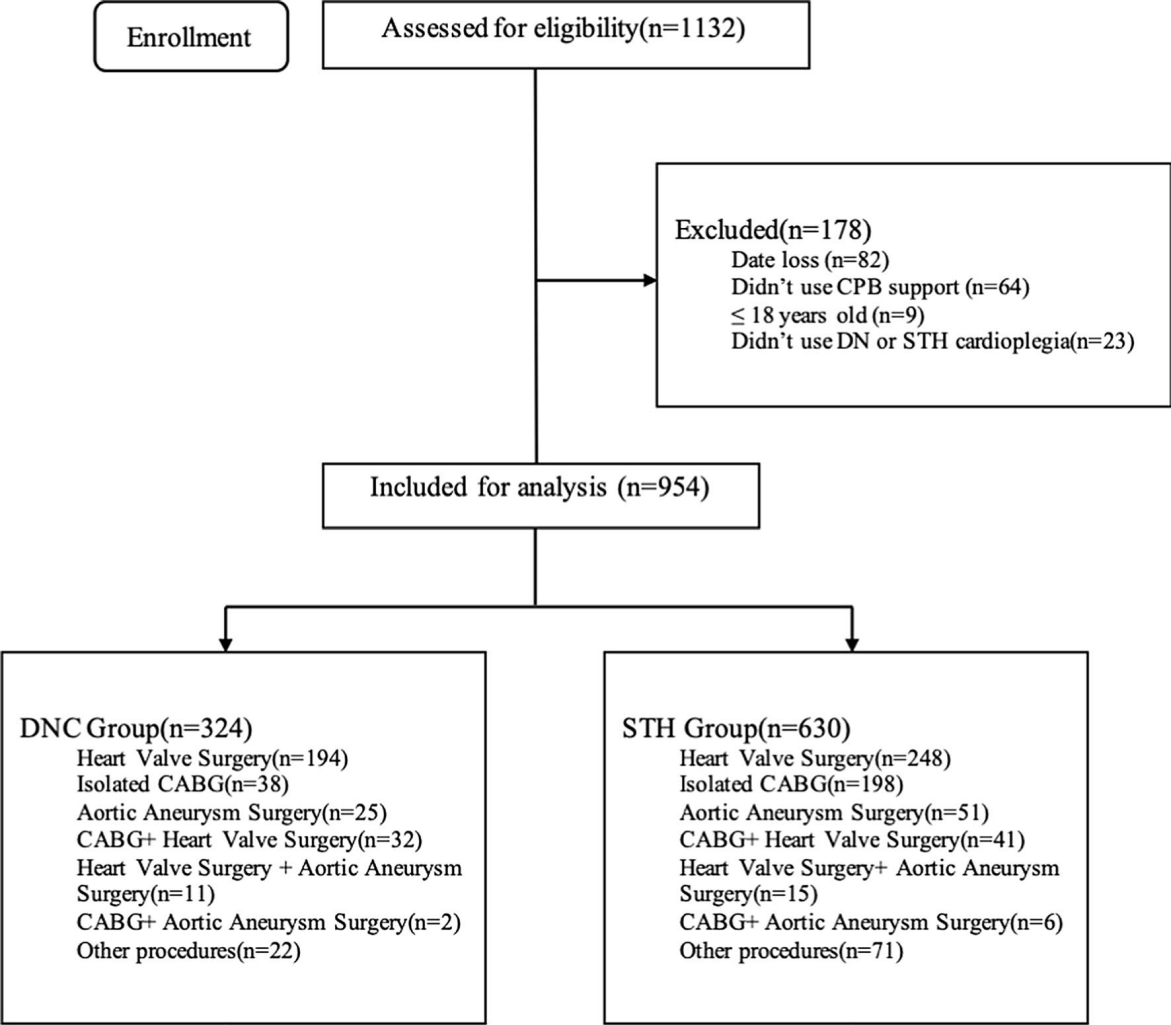
Consort flowchart. DNC group = Del Nido cardioplegia group; STH group = St. Thomas’s cardioplegia group;CABG = coronary artery bypass grafting

**Fig. 2:**
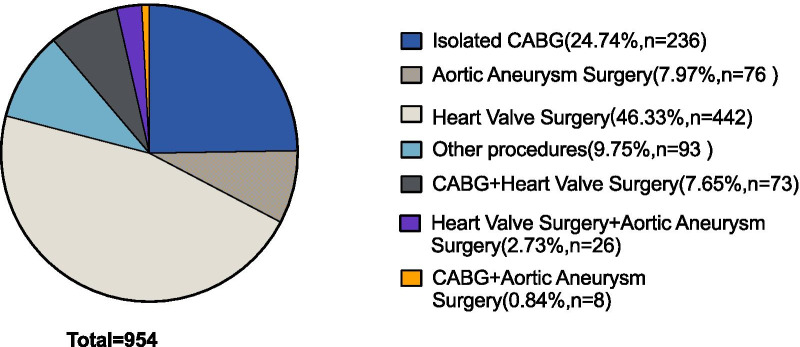
Classification of patients by type of surgery. “Other procedures” includes cardiac procedures not classified in the pie chart (i.e., surgical correction of congenital heart disease such as repair of atrial septal defect, atrial myxoma resection, cardiac transplantation, etc.). CABG = coronary artery bypass grafting

Among the 954 cases, patients were initially classified according to the type of cardiac surgery. A total of 442 patients received heart valve surgery, with 194 patients in DNC group and 248 patients in STH group. 236 patients received coronary artery bypass grafting (CABG), including 38 cases in DNC group and 198 cases in STH group.76 cases received aortic aneurysm surgery, wherein 25 cases in DNC group and 51 cases in STH group. A total of 93 cases were classified as other categories due to the small sample size, including surgical correction of congenital heart diseases such as repair of atrial septal defect, atrial myxoma resection, cardiac transplantation and other procedures. As for the combined surgery: 73 patients received coronary artery bypass surgery and heart valve surgery, with 32 in DNC group and 41 in STH group; 26 patients received heart valve surgery and aortic aneurysm surgery, with 11 in DNC group and 15 in STH group; and 8 patients received coronary artery bypass surgery and aortic aneurysm surgery, including 2 cases in DNC group and 6 cases in STH group. Further details were displayed in Figs. 1 and 2.

### Baseline characteristics of the patients in two groups

Of the 954 patients included in the study, 324 patients were in DNC group and 630 patients were in STH group. The average age (58.96 ± 13.26 vs. 60.44 ± 11.40, *P* = 0.074) and sex ratio (168/156 vs. 365/264, *P* = 0.080) of the patients in DNC group and STH group showed no statistical difference. In terms of disease severity, there were no statistical differences between APACHE II (*P* = 0.870) and euroSCORE (*P* = 0.497) of the patients in two groups. The main combined disease in DNC group and STH group was hypertension (*P* = 0.331), with no statistical difference. The proportion of patients with diabetes in DNC group was lower than that in STH Group (*P* < 0.05). There was no statistical difference in preoperative EF level and blood creatinine in two groups. The incidence of preoperative atrial arrhythmia was 5.25% vs. 4.76%, *P* = 0.865 and 2.78% vs. 2.35%, *P* = 0.879, respectively, and there was no statistical difference between two groups. Therefore, the perioperative state of the patients in DNC group and STH Group was similar. The baseline characteristics of the two groups of patients were shown in Table 1.

**Table1:** Characteristics of the patients

	DNC group (n = 324)	STH Group (n = 630)	Statistic	*P* value
AGE (year)	60.44 ± 11.40	58.96 ± 13.26	1.791^△^	0.074
Male (n, %)	168 (51.85%)	365 (57.94%)	3.064^※^	0.080
APACHE II	12.36 ± 5.16	12.29 ± 7.63	− 0.164^△^	0.870
euroSCORE	4.15 ± 1.26	4.00 ± 1.02	0.679^△^	0.497
pre-EF (%)	59.73 ± 7.52	59.83 ± 9.89	− 0.164^△^	0.870
pre-Cr (μmoI/L)	71.00 (59.00,87.25)	70.30 (59.00,87.00)	− 0.359^#^	0.720
Diabetes (n,%)	44 (13.60%)	120 (19.05%)	4.117^※^	0.043^*^
Hypertensive (n,%)	151 (46.60%)	316 (50.16%)	0.944^※^	0.331
Cerbral Infarction (n,%)	36 (11.11%)	57 (9.05%)	0.814^※^	0.367
Chronic Renal Insufficiency (n,%)	33 (10.19%)	53 (8.41%)	0.618^※^	0.365
pre-Atrial Fibrillation (n,%)	17 (5.25%)	30 (4.76%)	0.029^※^	0.865
pre-Ventricular Arrhythmia (n,%)	9 (2.78%)	15 (2.35%)	0.023^※^	0.879
OP time(hrs)	4.08 (3.50,4.67)	3.58 (3.00,4.33)	− 5.942^#^	< 0.001^**^
AB time(min)	80.00 (62.00,101.25)	61.00 (46.80,83.30)	− 8.607^#^	< 0.001^**^
CBP time(min)	116.00 (92.00,141.00)	89.00 (69.00,118.00)	− 8.713^#^	< 0.001^**^
K^+^_max_ (mmol/L)	4.92 ± 0.60	4.84 ± 0.54	2.136^△^	0.033^*^
post-EF (%)	60.00 (51.30,62.00)	60.00 (54.75,62.00)	− 1.224^#^	0.221
post-Cr _max_(umol/L)	88.45 (73.18,119.00)	87.60 (70.70,122.50)	− 0.735^#^	0.463
TnT(ng/mL)	366.80 (224.65,678.70)	274.45 (135.50,511.08)	− 5.142^#^	< 0.001^**^
CK-MB (U/L)	45.00 (31.00,64.00)	35.00 (24.00,55.00)	− 5.289^#^	< 0.001^**^
CK (U/L)	581.50 (438.00,881.00)	563.00 (391.50,802.00)	2.196^#^	0.028^*^
TnI (ng/mL)	0.59 (0.17,1.98)	0.42 (0.17,1.34)	− 1.851^#^	0.064
NT-proBNP (pg/mL)	799.96 (290.94,2204.76)	676.35 (242.45,1916.71)	− 1.733^#^	0.083
Lac(mmol/L)	2.70 (1.80,3.90)	2.50 (1.80,3.60)	− 1.572^#^	0.116
post-Atrial Fibrillation (n,%)	56 (17.39%)	100 (15.94%)	0.311^※^	0.577
post-Ventricular Arrhythmia (n,%)	40 (12.42%)	109 (17.40%)	4.224^※^	0.040^*^
Length of ICU stay(day)	1.97 ± 1.49	2.26 ± 1.46	− 2.864^△^	0.004^**^

Definitions: APACHE II = Acute Physiology and Chronic Health Evaluation II, euroSCORE = European system for cardiac operative risk evaluation, pre-EF = Perioperative ejection fraction, pre-Cr = Perioperative creatinine. ^△^. indicates that the comparison of variables between groups uses the t test, and the statistic provides a t value; ^※^. indicates that the comparison of variables between groups uses the chi-square test, the statistic provides an $\chi$ ^2^ value; ^#^. indicates that the comparison of variables between groups uses the sum test, and the statistic provides a Z value

### Comparison of postoperative data between the two groups

Compared with the patients in STH Group, the duration of operation, cardiopulmonary bypass (CBP)time, aortic cross clamp (AB) time of those in DNC group were much longer (*P* < 0.05). In terms of postoperative biochemical indexes, the highest blood potassium (K^+^_max_), maximum creatinine (Cr_max_), and blood lactate acid in the first 24 h after the surgery showed no significant difference between the two groups. However, the serum levels of CK, CK-MB, TnT were much higher in the patients of DNC group than those in STH group (*P* < 0.05) while the level of TnI in the two groups were parallel (*P* = 0.064).As for other cardiac function evaluation indexes, there was no significant difference in postoperative EF value and NT-proBNP between the two groups, suggesting that the two groups had similar levels of postoperative cardiac function. The incidence of postoperative ventricular arrhythmias in DNC group was 12.4%, notably lower than that in STH Group (17.4%, *P* = 0.040). Similarly, in DNC group, the length of ICU stay was also significantly shorter than that of STH Group (1.97 ± 1.49 vs. 2.26 ± 1.46, *P* < 0.001). Further indicators of the two groups were shown in Table 1.

### Logistic regression analysis for postoperative ventricular arrhythmia

Variables with statistical differences between the two groups were selected for multivariate logistic analysis for ventricular arrhythmia. The median value of operation time, CBP and AB time were 3.75 h,100 min and 69 min. Variables longer than the corresponding median time were categorized as prolonged.

Hyperkalemia was defined as the maximum blood potassium higher than 5.5 mmol/L in the first 24 h after surgery. According to the KDIGO guidelines [9], postoperative serum creatinine increase over 26.5umol/L was classified as acute kidney injury(AKI). Because of myocardial injury during operation, all the myocardial enzymes, including TnT, CK-MB, CK, TnI and NT-proBNP, had varying degrees of ascensions. All of them greatly exceeded the upper limit of normal range and were not accordant to normal distribution, so we use the corresponding median value (TnT > 309.2 ng/mL; TnI > 0.47 ng/mL; CK > 565 U/L; CK-MB > 38 U/L; NT-proBNP > 714.63 pg/mL) as the cut-off value to categorize these continuous variables. According to EuroSCORE, left ventricular systolic function (LVEF) was ranked as poor, moderate, and good corresponding to LVEF < 30%, 30% ≤ LVEF < 50%, LVEF ≥ 50%, and each rank were assigned to value 0, 1, 2 in the logistic model. The final results showed that DNC could significantly reduce the incidence of postoperative ventricular arrhythmias (OR = 0.479, 95% CI = 0.268–0.834, *P* = 0.011). More details in Table 2.

**Table 2:** Multivariate logistic regression for postoperative ventricular arrhythmia

Variable	B	S.E	OR	95% CI for OR		*P* value
				Lower	Upper	
Age	− 0.012	1.195	0.988	0.967	1.010	0.274
Gender	0.074	0.075	1.077	0.634	1.842	0.784
prolonged OP time	1.097	11.423	2.994	1.604	5.746	< 0.001^**^
prolonged CBP time	0.716	2.893	2.045	0.897	4.683	0.089
prolonged AB time	− 0.886	5.470	0.412	0.194	0.861	0.019^*^
Hyperkalemia	0.472	1.912	1.603	0.812	3.107	0.167
AKI	1.593	35.306	4.921	2.920	8.375	< 0.001^**^
increased TnT	− 0.016	0.002	0.984	0.516	1.874	0.960
increased TnI	0.081	0.094	1.084	0.646	1.821	0.759
increased CK	− 0.217	0.552	0.805	0.451	1.424	0.458
increased CK-MB	0.177	0.285	1.193	0.623	2.291	0.594
increased NT-proBNP	0.608	4.594	1.837	1.059	3.234	0.032^*^
DNC	− 0.736	6.499	0.479	0.268	0.834	0.011^*^
EF level	− 0.694	9.871	0.499	0.324	0.772	0.002^**^

Definitions: prolonged OP time = operation time > 3.75 h, prolonged CBP time = cardiopulmonary bypass time(CBP) > 100 min,prolonged AB time = aortic cross clamp time(AB) > 69 min,AKI = acute kidney injury, increased TnT/TnI/CK/CK-MB/NT-proBNP = TnT > 309.2 ng/mL, TnI > 0.47 ng/mL, CK > 565 U/L, CK-MB > 38 U/L, NT-proBNP > 714.63 pg/mL, LVEF < 30%, 30% ≤ LVEF < 50%, LVEF ≥ 50% were assigned to value 0, 1, 2

### Propensity score-based analysis for postoperative ventricular arrhythmia

**Fig. 3:**
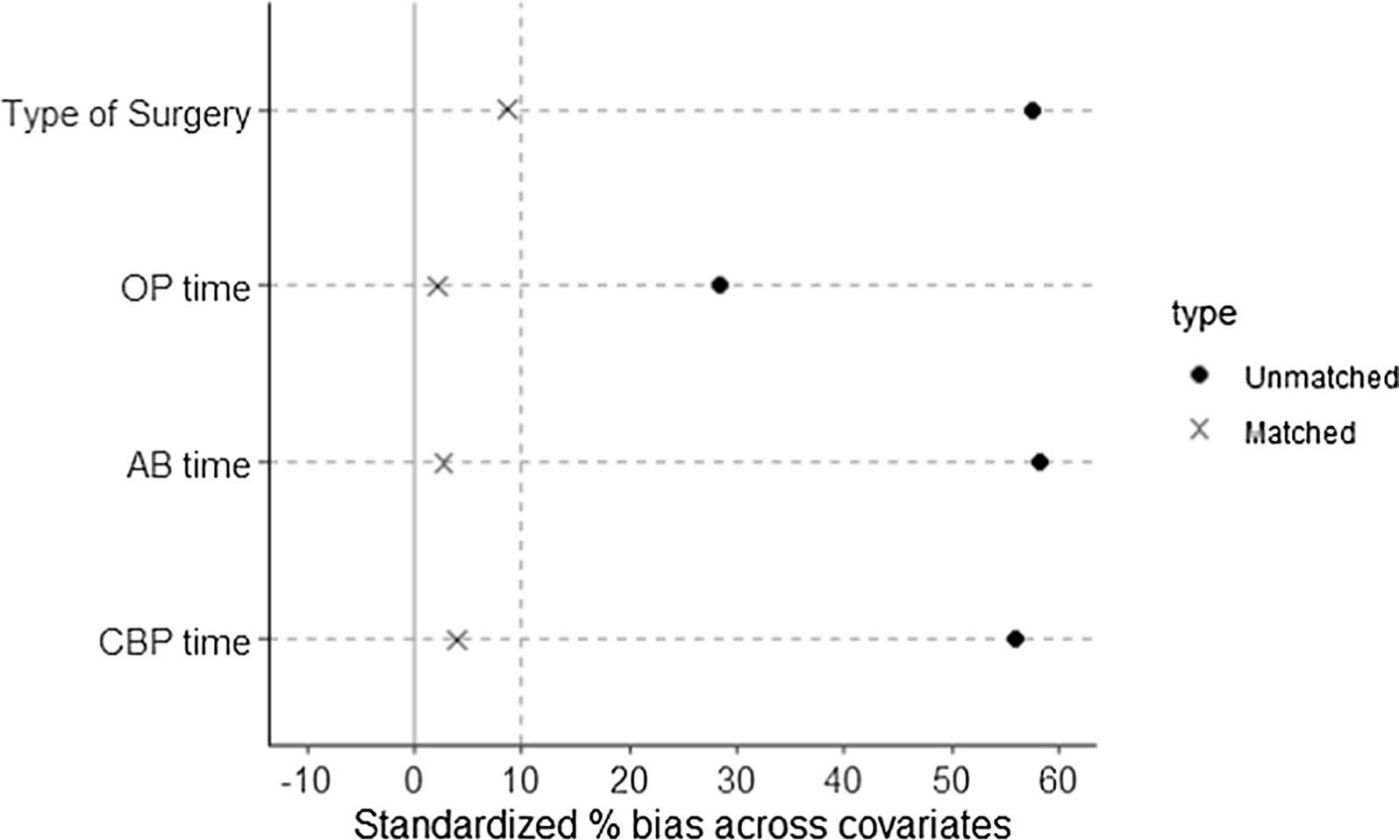
Standardized bias (%) across covariates before and after propensity score matching. The result showed that candidate covariates were well matched

There are large gaps in the surgical procedures performed between STH and DNC groups, including operation type, operation times, AB time and CBP time. So we performed a propensity score-based analysis to rule out the effect of surgical procedures on postoperative ventricular arrhythmia. DNC group patients (n = 163) were 1:1 propensity matched to STH group patients (n = 163) based on type of surgery, operation time, CBP time and AB time (Table 3). The balance of covariates was shown in Fig. 3, which demonstrates that these variables are well balanced after matching. After the 1:1 propensity matching, surgical procedure variables (including type of surgery, OP time, AB time and CBP time) were adequately balanced between groups. There were also no statistical differences on some postoperative variables such as K^+^_max_, post-EF, post-Cr_max_, TnT, CK-MB, CK, TnI, NT-proBNP, Lac and incidence of atrial fibrillation. However, the incidence of ventricular arrhythmia in DNC group was significantly lower in comparison with STH group (14.7% vs. 25.2%, *P* = 0.027) and the length of ICU stay was also significantly shorter than that in STH group (1.97 ± 1.58 vs. 2.55 ± 2.36, *P* = 0.010).

**Table 3:** Characteristics of the propensity-matched cohort

	DNC group (n = 163)	STH group (n = 163)	Statistic	*P* value
Type of Surgery				0.878
Heart Valve Surgery	95 (58.28%)	93 (57.06%)		
Isolated CABG	20 (12.27%)	14 (8.59%)		
Aortic Aneurysm Surgery	15 (9.20%)	16 (9.82%)		
CABG + Heart Valve Sugery	17 (10.43%)	18 (11.04%)		
Heart Valve Surgery + Aortic Aneurysm Surgery	6 (3.68%)	7 (4.29%)		
CABG + Aortic Aneurysm Surgery	2 (1.23%)	2 (1.23%)		
Other procdures	8 (4.91%)	13 (7.98%)		
OP time(hrs)	4.15 ± 1.01	4.13 ± 1.28	− 0.199^△^	0.843
AB time(min)	75.00 (58.50,90.50)	73.00 (57.50,91.50)	0.577^#^	0.564
CBP time(min)	111.00 (90.50,130.00)	106.00 (85.00,134.00)	− 0.802^#^	0.422
K^+^_max_ (mmol/L)	4.91 ± 0.54	4.91 ± 0.54	− 0.064^△^	0.949
post-EF (%)	60.00 (50.50,62.00)	59.00 (48.50,61.00)	− 1.119^#^	0.263
post-Cr _max_(umol/L)	93.00 (75.85,122.50)	91.00 (72.65,136.50)	− 0.585^#^	0.558
TnT(ng/mL)	352.70 (221.75,582.65)	334.50 (180.85,603.65)	− 0.563^#^	0.573
CK-MB (U/L)	44.00 (30.00,61.50)	41.00 (26.50,57.00)	− 0.954^#^	0.340
CK (U/L)	573.00 (438.00,865.50)	600.00 (405.50,855.50)	− 0.091^#^	0.927
TnI (ng/mL)	0.57 (0.17,1.52)	0.60 (0.22,1.61)	− 0.641^#^	0.522
NT-proBNP (pg/mL)	1013.31 (293.29,2425.86)	1061.30 (350.55,3060.15)	− 0.500^#^	0.617
Lac(mmol/L)	2.90 (1.85,3.90)	2.80 (2.00,3.65)	− 0.152^#^	0.879
Atrial Fibrillation (n, %)	34 (20.86%)	37 (22.70%)	0.072^※^	0.788
Ventricular Arrhythmia (n, %)	24 (14.72%)	41 (25.15%)	4.919^※^	0.027
Length of ICU stay(day)	1.97 ± 1.58	2.55 ± 2.36	2.584^△^	0.0103

Type of Surgery between groups was compared with Fisher’s Exact Test without statistic reported

Abbreviations as in Table 1

Subgroup analysis for patients with prolonged AB time.

To order to rule out the effect of AB time on myocardial injury, a subgroup analysis was performed on patients with prolonged AB time (longer than median time, 69 min). Finally, 204 patients in DNC group and 221 patients in STH Group were included in this subgroup analysis. The perioperative baseline data of the two groups had no statistical difference. The results revealed that none of the myocardial enzymes including TnT, CK-MB, CK, TnI and NT-proBNP showed statistical differences between the patients of the two subgroups. Comparatively, the incidence of ventricular arrhythmia (11.3% vs. 28.9%, *P* < 0.001) and length of ICU stay (2.06 ± 1.48 vs. 2.91 ± 1.51, *P* < 0.001) were significantly lower in DNC group than those in STH Group, suggesting that DNC may have a protective effect on myocardium (Table 4).

**Table 4:** Subgroup analysis for patients with prolonged AB time

	Sub-DNC group (n = 204)	Sub-STH Group (n = 221)	Statistic	*P* value
OP time(hrs)	4.38 (3.92, 5.08)	4.25 (3.67, 5.17)	− 1.091^#^	0.275
AB time(min)	93.50 (81.00, 110.25)	88.00 (76.00, 105.00)	− 2.513^#^	0.012^*^
CBP time(min)	130.00 (115.50, 150.00)	126.47 (110.00, 153.25)	− 1.443^#^	0.149
post-EF (%)	59.00 (50.00, 61.00)	60.00 (57.35, 61.00)	− 0.269^#^	0.788
TnT(ng/mL)	451.50 (272.20, 845.00)	421.80 (278.10, 981.35)	− 0.227^#^	0.820
CK-MB (U/L)	53.00 (36.00, 67.00)	51.00 (34.75, 72.00)	− 0.323^#^	0.747
CK (U/L)	677.50 (472.00, 961.00)	652.00 (476.00, 972.00)	− 0.066^#^	0.947
TnI (ng/mL)	0.78 (0.18, 2.50)	0.85 (0.26, 2.50)	− 0.763^#^	0.446
NT-proBNP (pg/mL)	930.75 (390.18, 2237.65)	850.71 (308.52, 2610.84)	− 0.030^#^	0.976
Lac(mmol/L)	2.80 (2.10, 3.90)	2.70 (1.90, 4.10)	− 0.273^#^	0.785
Atrial Fibrillation (n,%)	37 (18.14%)	46 (20.81%)	0.484^※^	0.487
Ventricular Arrhythmia (n,%)	23 (11.27%)	64 (28.89%)	13.118^※^	< 0.001^**^
Length of ICU stay(day)	2.06 ± 1.48	2.91 ± 1.51	− 5.848^△^	< 0.001^**^

Abbreviations as in Table 1

## Discussion

At present, there is no clear consensus on the safety and effect of DNC on various cardiac surgery. Our study showed that DNC may reduce the incidence of postoperative ventricular arrhythmias. Results from our study showed that the total incidence of ventricular arrhythmias in DNC group was 12.4%, notably lower than that in STH Group (17.4%, *P* = 0.040). Amatya A and Sadr-Ameli MA reported that the incidence of ventricular tachyarrhythmia after cardiac surgery in Asian people was 24.4% to 26.6% which was similar to our study[10, 11]. Multivariate logistic regression analysis showed that DNC could be an independent protection factor against the incidence of postoperative ventricular arrhythmias (OR = 0.479, 95% CI = 0.268–0.834, *P* = 0.011). So far, we did not find similar research results, possible reasons are speculated as follows.

### Protective mechanism of DNC solution

Cardioplegia will increase the concentration of extracellular potassium and reduce the potential difference between the inside and outside potassium of the cell. Sodium (Na^+^) cannot flow inwards so the heart stops in diastole. At depolarized potentials, some sodium currents remain active as “window” currents, resulting in abnormal ionic gradients of sodium and calcium, intracellular calcium overload, and thus inhibit myocardial recovery [12]. The protective mechanism of DNC solution might be lidocaine, a concurrent sodium channel blockade which can increase the refractory period of the cardiac myocyte, and the calcium antagonist action of magnesium. Furthermore, hypertonic mannitol can clear free radicals and as well reduce myocardial cell swelling [13], beneficial for the transmission of sinus rhythm.

However, this study showed that myocardial enzymes such as the serum levels of CK-MB, CK, TnT and TnI increased in DNC group compared with STH group. The reason may be related to the myocardial injury caused by long operation time and long aortic cross clamp time in DNC group. A study from Charette K [14] has confirmed that there is no difference in the 90-min plus arm of the study between DNC and STH Group when comparing risk adjustment for congenital heart surgery (RACHS) (*P* = 0.6), CPB time (*P* = 0.5), AB time (*P* = 0.5).In our opinion, among CPB, AB and OP time, AB time represents myocardial ischemia time, which may most affect the degree of myocardial injury. In order to exclude the differences caused by the prolonged AB time, we also performed a subgroup analysis in patients with prolonged AB time. The subgroup analysis results suggested that there were no statistical differences in myocardial enzymes between two subgroups.

### DNC solution reduce the incidence of postoperative ventricular arrhythmias

As mentioned earlier, DNC solution contains lidocaine and mannitol. The incidence of postoperative ventricular arrhythmias in DNC group was still markedly lower than STH Group (11.3% vs. 28.9%, *P* < 0.001, Table 4).

Either incidence of preoperative atrial arrhythmia or ventricular arrhythmia showed no difference between the two groups, and were lower than other studies. This may occur due to incomplete preoperative data, such as differences in medical history accuracy caused by different education levels of patients. ECG data at admission cannot fully represent whether the patient has an arrhythmia history, the incidence of preoperative arrhythmia may be underestimated.

In our study serum potassium of DNC and STH group seems to be different, but both of them were within normal range and the gap between them was small. We cannot find any research to confirm the difference of potassium could result to the incidence of postoperative ventricular arrhythmias. Although the serum potassium of the two groups were nearly equal in the Propensity score-based matched cohort (Table 3), the incidence of postoperative ventricular arrhythmias was still lower in DNC group (14.72% vs.25.15%, *P* = 0.027). Our ICU department regularly monitors patients’ serum potassium and gives exogenous chloratum kalium supplementation. Therefore, the difference of potassium level was not caused by the type of cardioplegia, and could not positively affect the incidence of postoperative ventricular arrhythmias.

### DNC solution for complicated cardiac surgery

Single-dose of DNC perfusion can maintain 90 min, reducing the number of repeated perfusions and the number of intraoperative interventions. Studies from O’Donnell [15] and Yerebakan [16] found that the use of DNC solution was associated with shorted aortic cross clamp times. A meta-analysis from Ivancarmine Gambardella [17] proved that DNC solution reduced ischemic time, CPB time, reperfusion fibrillation and cardiac enzymes compared with multidose cardioplegia.

This characteristic will allow surgeons to streamline the workflow of procedures requiring cardioplegic arrest. DNC can improve efficiency and increase the tolerance of myocardial ischemia to avoid reperfusion disadvantages [18]. Indicating that DNC may have better myocardial protective effect than STH and may be more suitable for patients with complicated cardiac surgery.

Using DNC in adult cardiac surgery patients currently lacks support from strong medical evidence. The Cleveland Clinic does not recommend using DNC solution for protecting the ischemic myocardium such as patients with coronary artery disease [8]. As DNC is a potassium-loaded cardioplegia, adequate and uniform distribution of cardioplegia solution to the myocardium will not be guaranteed when the patient has either large-vessel disease or dysfunction of microcirculation. Residual potassium in the coronary vessels can predispose to coronary vasoconstriction and exacerbate myocardial ischemia during reperfusion, with the accompanying conduction anomalies [19].

On the opposite, we conducted a propensity score-based analysis to reduce surgical procedures’ bias between groups. Our research data suggested that there was still no significant difference in OP time, AB time and CBP time, and the ventricular arrhythmia, and length of ICU stay were still statistically different between cohorts (Table 3), suggesting that DNC has the same protecting effects towards myocardial on overall cardiac function and could be safely used in all cardiac surgeries. At the same time, studies from Gustavo E [20] and Christian O’Donnell’s [15] have also confirmed the safety of DNC in coronary artery bypass surgery. When DNC compared with blood cardioplegia, there were no differences in the baseline characteristics nor any differences in the 30-day incidences of myocardial infarction (0% in both groups), all-cause death (0% in both groups), and ICU stay (2.9 ± 2.5 vs. 2.9 ± 4.6, *P* = 0.922) [20]. It seems that DNC provides a safe, economic and efficient way to perform on-pump CABG surgery in low-risk patients. However, we still strongly recommend that safety of using DNC in coronary bypass surgery require validation using large-sample datasets.

### DNC solution and outcomes

Data of our study suggested a shorter length of ICU stay in the patients of DNC group than those of STH group, which may be related to the reduced incidence of ventricular arrhythmia in DNC group. Patients in DNC group were more likely to have stable hemodynamics and can conduct a spontaneous breathing test (SBT) as soon as possible and then enter the extubation process quickly.

The study of DNC solution from Schutz [21] confirmed that the overall infection rate and sternum infection rates in patients with DNC were significantly lower (0.6% vs 3.3%, *P* = 0.027). One potential mechanism may be related with the lower peak glucose level in DNC group than that in STH Group (168 vs. 201 mg/dL, *P* < 0.0001). As DNC does not require additional glucose, which could help to control the perioperative blood glucose of the patients [22] and decrease the possibility of postoperative hyperglycemia, and thereby reduce the incidence of infection and shorten ICU stay.

In conclusion, DNC can effectively reduce the incidence of ventricular arrhythmia, reduce the length of ICU stay, reduce perfusion frequency, and increase efficiency, is a new alternative choice in cardiovascular surgery.

### Limitations

This study focused on patients underwent cardiac surgery throughout one year, especially on the analysis of patients' vital signs and examination indicators when entering ICU for further treatment. Firstly, they were not grouped according to the type of surgery procedures. The pathophysiological differences among heart valve surgery, coronary artery bypass surgery, and aortic aneurysm surgical patients and other procedures cannot be clearly distinguished. Whether DNC is more beneficial for certain patients may need further study. Secondly, this study used myocardial enzymes and cardiac ultrasound EF value to evaluate the patients’ cardiac function, which may not be comprehensive enough. It may be more conducive to include postoperative vasoactive drug dose, application of mechanical assist device such as intra-aortic balloon pumping (IABP), extracorporeal membrane oxygenation (ECMO). Thirdly, we didn’t compare the data of new-onset ventricular arrhythmia and new-onset atrial arrhythmia between two groups of patients because of the concern of preoperative arrhythmia baseline bias. In the future, patients with accurate basic arrhythmia data can be selected for comparison. Last but not least, this study was a retrospective study. More prospective studies such as randomized controlled trial (RCT) and cohort analysis are needed to further verify the results.

## Conclusions

Del Nido cardioplegia may help to reduce the incidence of postoperative ventricular arrhythmias, shorten the length of ICU stay, and improve the prognosis of the patients with cardiac surgery.

## Data Availability

The datasets used and/or analysed during the current study available from the corresponding author on reasonable request.

## Abbreviations

DNC: Del Nido cardioplegia
CPB: cardiopulmonary bypass
ICU: Intensive Care Unit
OPCAB: off-pump coronary artery bypass graft
APACHE II: Acute Physiology and Chronic Health Evaluation II
euroSCORE: European system for cardiac operative risk evaluation score
EF: ejection fraction
AB: aortic occlusion
TnT: troponin T
TnI: troponin I
CK: creatine kinase
CK-MB: creatine kinase isoenzyme
NT-proBNP: N-terminal forebrain natriuretic peptide precursor
PSM: propensity score matching
CABG: coronary artery bypass grafting
AKI: acute kidney injury
LVEF: left ventricular systolic function
SBT: spontaneous breathing test
IABP: intra-aortic balloon pumping
ECMO: extracorporeal membrane oxygenation
RCT: randomized controlled trial

## References

[1] Hendry PJ, Masters RG, Haspect A (1994). Is there a place for cold crystalloid cardioplegia in the 1990s?. Ann Thorac Surg.

[2] Ali JM, Miles LF, Abu-Omar Y, Galhardo C, Falter F (2018). Global cardioplegia practices: results from the global cardiopulmonary bypass survey. J Extra-Corporeal Technol.

[3] Matte GS, del Nido PJ (2012). History and use of del Nido cardioplegia solution at Boston Children's Hospital. J Extra-Corporeal Technol.

[4] Govindapillai A, Hancock Friesen C, O'Blenes SB (2016). Protecting the aged heart during cardiac surgery: single-dose del Nido cardioplegia is superior to multi-dose del Nido cardioplegia in isolated rat hearts. Perfusion.

[5] Ota T, Yerebakan H, Neely RC, Mongero L, George I, Takayama H, Williams MR, Naka Y, Argenziano M, Bacha E (2016). Short-term outcomes in adult cardiac surgery in the use of del Nido cardioplegia solution. Perfusion.

[6] Kim JS, Jeong JH, Moon SJ, Ahn H, Hwang HY (2016). Sufficient myocardial protection of del Nido cardioplegia regardless of ventricular mass and myocardial ischemic time in adult cardiac surgical patients. J Thorac Disease.

[7] Smigla G, Jaquiss R, Walczak R, Bonadonna D, Kaemmer D, Schwimer C, Lodge A (2014). Assessing the safety of del Nido cardioplegia solution in adult congenital cases. Perfusion.

[8] Kim K, Ball C, Grady P, Mick S (2014). Use of del Nido cardioplegia for adult cardiac surgery at the cleveland clinic: perfusion implications. J Extra-Corporeal Technol.

[9] Khwaja A (2012). KDIGO clinical practice guidelines for acute kidney injury. Nephron Clin Pract.

[10] Amatya A, Sharma A, Pokharel JN, Amatya A, Shrestha SM (2015). Ventricular tachyarrhythmia after aortic cross clamp release in cardiac surgeries. J Nepal Health Res Counc.

[11] Sadr-Ameli MA, Alizadeh A, Ghasemi V, Heidarali M (2013). Ventricular tachyarrhythmia after coronary bypass surgery: incidence and outcome. Asian Cardiovasc Thorac Ann.

[12] Chambers DJ, Fallouh HB (2010). Cardioplegia and cardiac surgery: pharmacological arrest and cardioprotection during global ischemia and reperfusion. Pharmacol Ther.

[13] Powell WJ, Dibona DR, Flores J, Leaf A (1976). The protective effect of hyperosmotic mannitol in myocardial ischemia and necrosis. Circulation.

[14] Charette K (2012). Commentary on: Ninety minutes and longer: single dose myocardial protection technique utilizing the Del Nido cardioplegia solution for myocardial protection during congenital heart surgery procedures. Perfusion.

[15] O'Donnell C, Wang H, Tran P, Miller S, Shuttleworth P, Boyd JH (2019). Utilization of Del Nido cardioplegia in adult coronary artery bypass grafting - a retrospective analysis. Circ J.

[16] Yerebakan H, Sorabella RA, Najjar M, Castillero E, Mongero L, Beck J, Hossain M, Takayama H, Williams MR, Naka Y (2014). Del Nido cardioplegia can be safely administered in high-risk coronary artery bypass grafting surgery after acute myocardial infarction: a propensity matched comparison. J Cardiothorac Surg.

[17] Gambardella I, Gaudino MFL, Antoniou GA, Rahouma M, Worku B, Tranbaugh RF, Nappi F, Girardi LN (2020). Single- versus multidose cardioplegia in adult cardiac surgery patients: a meta-analysis. J Thorac Cardiovasc Surg.

[18] Ginther RM, Gorney R, Forbess JM (2013). Use of del Nido cardioplegia solution and a low-prime recirculating cardioplegia circuit in pediatrics. J Extra-Corporeal Technol.

[19] Dobson GP (2010). Membrane polarity: a target for myocardial protection and reduced inflammation in adult and pediatric cardiothoracic surgery. J Thorac Cardiovasc Surg.

[20] Guajardo Salinas GE, Nutt R, Rodriguez-Araujo G (2017). Del Nido cardioplegia in low risk adults undergoing first time coronary artery bypass surgery. Perfusion.

[21] Schutz A, Zhang Q, Bertapelle K, Beecher N, Long W, Lee VV, Pan W, Arcaro M, Ghanta R, Jimenez E (2020). Del Nido cardioplegia in coronary surgery: a propensity-matched analysis. Interact Cardiovasc Thorac Surg.

[22] Timek T, Willekes C, Hulme O, Himelhoch B, Nadeau D, Borgman A, Clousing J, Kanten D, Wagner J (2016). Propensity matched analysis of del Nido cardioplegia in adult coronary artery bypass grafting: initial experience with 100 consecutive patients. Ann Thorac Surg.

